# Plasmonic Schottky photodetector with metal stripe embedded into semiconductor and with a CMOS-compatible titanium nitride

**DOI:** 10.1038/s41598-019-42663-3

**Published:** 2019-04-15

**Authors:** Jacek Gosciniak, Fatih B. Atar, Brian Corbett, Mahmoud Rasras

**Affiliations:** 1 New York University Abu Dhabi, Saadiyat Island, PO Box 129188, Abu Dhabi, UAE; 2 Tyndall National Institute, Lee Maltings, Cork, Ireland

## Abstract

Here we propose an original waveguide-integrated plasmonic Schottky photodetector that takes full advantage of a thin metal stripe embedded entirely into a semiconductor. The photodetector is based on the long-range dielectric-loaded surface plasmon polariton waveguide with a metal stripe deposited on top of a semiconductor rib and covered by another semiconductor. As the metal stripe is entirely surrounded by semiconductor, all hot electrons with appropriate k-vectors can participate in transitions that highly enhances the electron transfer, and consequently the internal quantum efficiency. In addition, a high coupling efficiency from the photonic waveguide to the photodetector is simulated exceeding 90 % which enhances the external quantum efficiency. Calculations show that a responsivity exceeding 0.5 *A*/*W* can be achieved at telecom wavelength of 1550 *nm* and the bandwidth can exceed 100 *GHz*. Furthermore, it is shown that titanium nitride is a perfect material for the photodetector as it provides a low Fermi energy and long electron mean free path that enhance the hot electron transfer to the semiconductor. In addition, it shows reasonable metallic behavior and CMOS compatibility. Measurements showed that the Schottky barrier height between titanium nitride and p-doped silicon reaches 0.69–0.70 *eV* that matches the optimum signal-to-noise ratio operation calculated at 0.697 *eV*.

## Introduction

Optical transceivers are the main building blocks of optical interconnects and consist of the laser light source, modulator, (de)multiplexer, and photodetector^1–3^. While the main task for the modulator is to encode the electrical signals into light propagating along the optical channel, the key principle of the photodetector is the conversion of absorbed photons back into electrical signals where information can be further processed^4^. Compared to the laser and modulator that can be combined into one device element as in, for example, directly modulated VCSELs, and thus avoided in the optical link, the photodetector is a crucial element that cannot be replaced. Furthermore, it is the last element in the optical link, thus it should operate efficiently under low input powers. As the optical energy received at the photodetector is directly related with the transmitter optical output power and the total link loss, it is crucial to minimize the optical losses at the photodetector. Another important issue in the realization of optical transceivers is the CMOS compatibility since photonics will have to be co-integrated with electronics^3^. Additional challenges are that small but slow electronic components have to be integrated with fast but large sized photonic elements. Plasmonics, where a metal electrode is part of the waveguide can serve as a bridge between photonics and electronics and provide components with sizes similar to electronics and speeds characterized by photonics^5,6^.

Photodetectors operate on the basis of the photoelectric effect or exhibit an electrical resistance dependent on the incident radiation^4,7^. The operation principal is based on the absorption of photons and the subsequent separation of the photogenerated charge carriers - electron-hole (e-h) pairs. They suffer, however, from low efficiency either because the near-infrared (NIR) photons energy at telecom wavelengths (0.79–0.95 *eV*) is not sufficient to overcome the Si bandgap (1.12 *eV*) or low detection area in the case of Ge-based photodetectors (bandgap 0.67 *eV*). Recently, a 100 GHz plasmonic Ge waveguide photodetector has been shown with an internal quantum efficiency reaching 36 % at a wavelength of 1310 *nm*^8^. However, as the wavelength increases to 1550 *nm*, the internal quantum efficiency and, as a consequence, the responsivity drops significantly. Very similar results were obtained for a compact photonic waveguide-integrated germanium-on-insulator (GOI) photodetector where an internal quantum efficiency of 39%, corresponding to the responsivity of 0.41 *A*/*W* at 1 *V* bias voltage, was measured^9^. For 1500 *nm* wavelengths however, the responsivity was measured at 0.14 *A*/*W* at 1 *V* corresponding to an internal quantum efficiency of 12%^8^. An alternative approach utilizes the intrinsic absorption of metal for photodetection that is accomplished by internal photoemission (IPE) in a Schottky diode^10–17^. In this configuration, photoexcited (“hot”) carriers from the metal are emitted into the semiconductor over a potential Φ_*B*_, called the Schottky barrier, that exists at the metalsemiconductor interface. To this date, the highest responsivity of 0.37 *A*/*W* at wavelength of 1550 *nm* and at a reverse bias voltage of 3 *V* was achieved in a metal-graphene-silicon plasmonic Schottky photodetector with inverse dielectric-loaded surface plasmon polariton (DLSPP) design^18^. The high responsivity was attributed to the combined effect of light absorption close to the graphene-silicon Schottky interface as well as enhanced injection from the graphene-silicon interface. At such a high voltage, avalanche multiplication of carriers takes place in graphene corresponding to a photogain of 2. In another design that is based on a metal-insulator-metal (MIM) arrangement, a responsivity of 0.12 *A*/*W* at 1550 *nm* for a bias voltage of 3.5 *V* and bandwidth exceeding 40 GHz was achieved^19^. However, this arrangement requires very advanced fabrication techniques related with a small gap of 75 *nm* between metals consisting of a plasmonic photodetector and preferable asymmetric metal electrodes arrangement. At the semiconductor, the injected carriers are accelerated by the electric field in the depletion region of the Schottky diode and then collected as a photocurrent at the external electrical contacts. Usually, the Schottky barrier is lower than the bandgas of most semiconductors thus allowing photodetection of NIR photons with energy *hν* > Φ_*B*_. The process of photo-induced emission of electrons from metals and their collection was described by Spicer^20,21^ based on Fowler’s proposal and consists of a three steps: (1) generation of hot electrons in the metal through the absorption of photons, (2) diffusion of a portion of the hot electrons to the metal-semiconductor/insulator interface before thermalization, and (3) injection of hot electrons with sufficient energy and the correct momentum into the conduction band of the semiconductor/insulator through internal photoemission.

To enhance the efficiency of the IPE process it is desirable to confine the optical power at the metal boundary with the materials forming the Schottky barrier. This will allow an increase in the interaction of light with the metal in very close vicinity of the interface where the photoemission process take place. The solution for this is well known and it is called the surface plasmon polariton (SPP). The SPP are guiding optical surface waves propagating along the boundary between metal and dielectric with the maximum field located in this interface and decaying exponentially in both media^5,6^. One of the main advantage of SPP relies in the fact that it is not diffraction limited and it enables a tight confinement of the optical field to subwavelength dimensions. The SPP offers a long interaction length between the propagating mode and the photodetector, thus allowing a larger portion of the optical energy to be absorbed nearby the Schottky barrier. Plasmonic photodetectors are suggested by ITRS as an alternative technology to overcome the foreseeable scaling limitations of conventional components^22^. It offers multiple advantages such as high integration densities, low device capacitance allowing for higher bandwidth operation, and ultra-low energies to operate.

Surface plasmon polaritons are electromagnetic surface waves which are coherently coupled to charge carrier density fluctuations on a metal and propagating at the interface between a metal and an insulator/semiconductor i.e., between materials with opposite signs of the real part of the permittivity^5,6^. The negative real part of the permittivity in metals is related to the collective motion of the conduction electrons, plasma oscillations. Analogously to the photon, they exhibit wave-like and particle-like behavior. However, compared to the photons, they are not diffraction limited and are able to support intense electromagnetic field concentration at the interface between metal and semiconductor/insulator. Surface plasmons can decay either radiatively via emission of photons or non-radiatively through the generation of excited carriers, so called hot carriers. These photo-excited hot carriers are able to overcome the potential barrier between metal and semiconductor/insulator, which leads to a light-induced charge separation and hence a measurable current. Furthermore, the potential barrier can be overcome either directly or through quantum mechanical tunneling effects with the probability dependent on the barrier width and height as well as the charge carrier energy.

## Results

**Figure 1:**
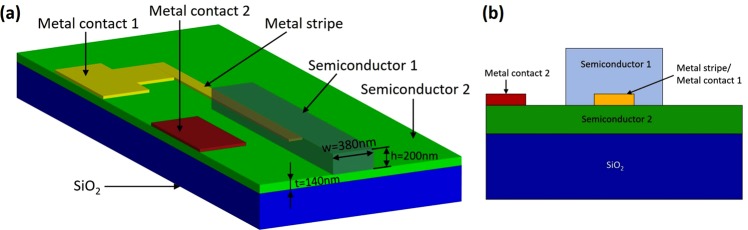
(**a**) Proposed LR-DLSPP photodetector arrangement and (**b**) cross-section of the structure.

The proposed photodetector arrangement consist of a metal stripe embedded in the semiconductor/insulator material (Fig. 1). For a metal stripe embedded in a dielectric there are two SPPs, one on each side of the metal-dielectric interfaces that are bound to the metal interface, and with the electromagnetic energy located partially in the metal and in the dielectric. The amount of the energy in the metal and dielectric depends on the material optical properties and waveguide geometry. The penetration depth of the electromagnetic field into the dielectric depends significantly on the permittivity of the semiconductor/insulator and the plasmonic waveguide configuration and it is typically on the order of *λ*/2. In contrast, the penetration depth of light into a metal, so called the skin depth, depends on the metal optical properties. Thus, a large negative real permittivity, that is a consequence of a larger plasma frequency due to larger carrier concentration, gives small penetration into the metal, while a small imaginary permittivity leads to lower losses. The skin depth in the metal is usually in the range of 10–20 *nm*. Based on this it can be deducted that field penetration into the metal influences the trade-off between confinement and propagation losses - the less light inside the metal and more inside the dielectric, the smaller the loss and smaller confinement. To reduce the absorption losses, the longitudinal component of the electric field in the metal that is responsible for the absorption losses has to be minimized. For the case of the metal stripe embedded into dielectric ridge it can be achieved by decreasing (reducing) the metal stripe thickness below the penetration depth of the SPP into the metal, so the two SPP modes associated with two opposite interfaces can overlap and form a new SPP wave with an increased propagation range, the so called long-range dielectric-loaded SPP (LR-DLSPP)^23–26^. In this case, the longitudinal component of the electric field in the metal is minimized. LR-DLSPP denotes here the plasmonic waveguide where the metal stripe is embedded inside a dielectric/semiconductor ridge that ensures good mode confinement (Fig. 1).

**Figure 2:**
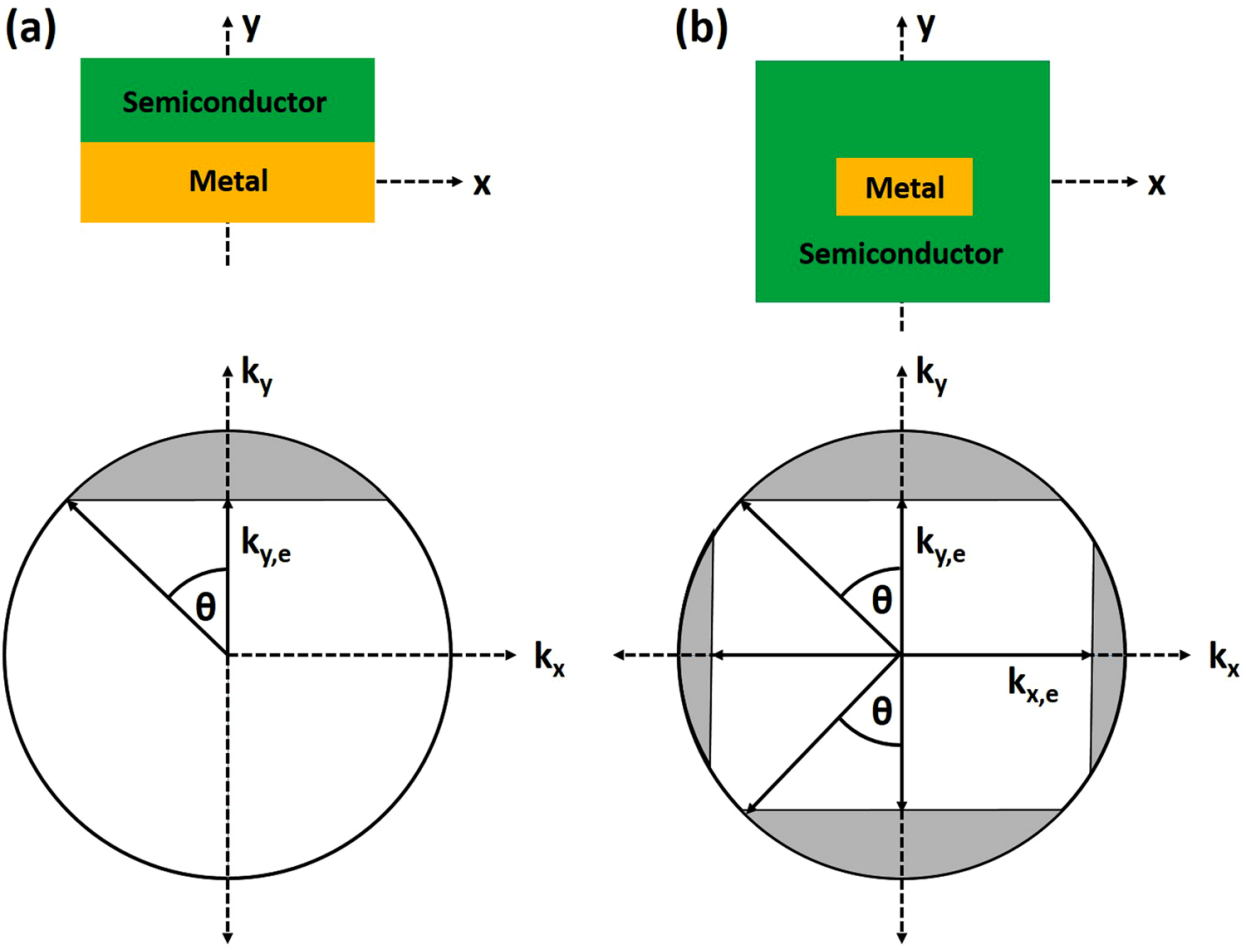
Metal - silicon Schottky barriers and k-space emission cone for (**a**) single interface and (**b**) metal stripe embedded into semiconductor.

To improve the transmission probability of hot carriers from metal to semiconductor we propose to place the metal stripe inside the semiconductor. Compared to the previously reported works where the metal-semiconductor-metal (MIM) waveguide was proposed^19,27^ or inverse-DLSPP waveguide with metal electrode on top of the Si waveguide^18,28–30^, in our proposed arrangement (Fig. 1) much more hot carriers will participate in transitioning to the semiconductor as the metal electrode is embedded inside the semiconductor. Here, as semiconductor 1 and semiconductor 2 (Fig. 1) we consider either n-doped Si or p-doped Si. However, depending on the requirements, any other semiconductor materials can be implemented for this design as long as the refractive index of both semiconductors are close to each other. The second electrode (Metal contact 2) is placed outside the waveguide in close proximity to the waveguide/photodetector (Fig. 1). It can be fabricated on either sides of the waveguide depending on requirements. For the thin metal stripe embedded within the semiconductor, the Schottky barrier exists on both sides of the metal-semiconductor interface, thus the probability of emission of photo-excited carriers is doubled due to the doubling of the interfaces^31^ (Fig. 2). Additionally, it is further increased due to multiple carrier reflections within the metal film.

In the MIM (Ti-Si-Au) arrangement^19^ most of the hot carriers will actually move in the opposite direction to the junction and after a short period of time will be thermalized. Furthermore, only a part of the SPP will be dissipated at the desired interface (Ti-Si) and participate in the hot carrier generation. The hot carriers generated in the opposite junction (Au-Si) will give rise to a dark current. In the inverse-DLSPP arrangements^18,28–30^, only a small fraction of hot carriers will participate in transitions to the semiconductor, as most of them will move in directions that do not give a rise to a photoemission process. Embedding the plasmonic stripe within the semiconductor boosts the hot electron transfer efficiencies by providing more momentum space for hot electron emission^32^ (Fig. 2). A plasmonic stripe embedded in the semiconductor forms a 3D Schottky barrier on all sides of the plasmonic stripe^32^ (Fig. 2) compared to the planar Schottky interface in the MIM^19^, inverse-DLSPP^18,28–30^ or metal stripe on semiconductor^33^ photodetector arrangements. It was experimentally shown^32^ that for the same number of photons absorbed by a metal stripe, the structures embedded in the semiconductor produce higher responsivity that can be directly attributed to an enhancement in charge injection over the additional Schottky barriers. Furthermore, a higher photocurrent enhancement for thinner stripes was observed indicating the major contribution of the ballistic hot electrons produced by plasmon decay. As presented in paper^32^, the embedded nanowires, under normal incidence have 25x greater efficiency than comparable planar Schottky devices suggesting that 3 *D* Schottky barriers can be a key design feature for increasing the efficiency of plasmon-based photodetectors.

The SPP propagating on each side of the plasmonic stripe embedded into semiconductor along the metal-semiconductor interface generates hot electrons in the very close vicinity of the Schottky interface. Because of the nature of the SPP, the electric field component is perpendicular to the interface that entails the generation of hot electrons with a momentum vector perpendicular to the Schottky interface. Furthermore, as it was recently demonstrated, the sharp corners of the metal stripe compress the SPPs producing hot electrons with high momentum perpendicular to the interface and thus further enhancing the photoemission efficiency^10^. In the LR-DLSPP arrangement proposed here, the highest electric field is localized in all four edge corners in order to enhance the photoemission process.

**Figure 3:**
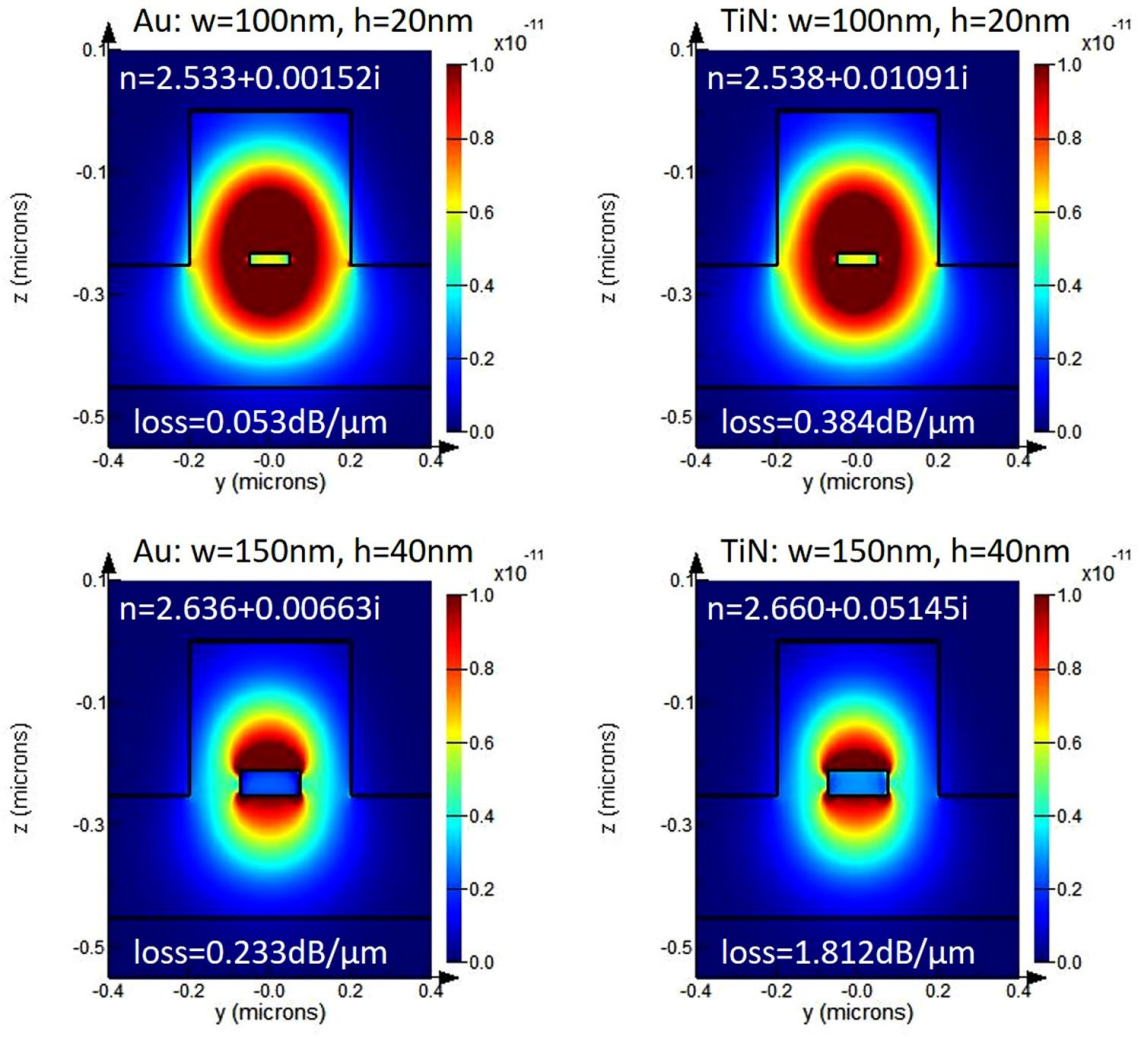
Mode effective index and calculated losses for LR-DLSPP waveguide with Au and TiN stripes embedded into Si for different stripe widths and thicknesses for the structure presented in Fig. 1

The main source of losses in the proposed arrangement are absorption losses by the metal stripe. To compare absorption losses and mode effective indices, two different metals (Au and TiN) were simulated, and we considered two different metal dimensions, i.e., *w* = 100 *nm*, *h* = 20 *nm*, and *w* = 150 *nm*, *h* = 40 *nm*. The ridge dimension and rib thickness were kept constant at *w* = 380 *nm*, *h* = 200 *nm* and *t* = 140 *nm* respectively. The same waveguide was used to couple the TM mode from a photonic waveguide to the photodetector. The mode effective index for such a waveguide was calculated at *n*_*eff*_ = 2.49. The power absorbed by the metal stripe generates hot electrons that can participate in transitions to the semiconductor (Si). Thus, the more power absorbed by the metal stripe, the more hot electron transfer. As it can be observed (Fig. 3), the thicker the metal stripe, the higher the absorption losses. However, the thicker the metal stripe, the higher the real part of the mode effective index resulting in higher coupling losses between the photonic waveguide and photodetector. In this way, as the stripe becomes thicker the internal quantum efficiency rise, however, the external quantum efficiency decreases due to the effect of lower coupling efficiency. Thus, special care has to be put on the proper choice of metal stripe dimensions. The absorption losses are higher for TiN compared to Au which is directly related with the real and imaginary part of the permittivities - TiN has a lower negative real part of permittivity and higher imaginary part that enhances absorption. For the same metal stripe dimensions, TiN provides over 7 times higher absorption compared to Au, and only slightly higher real part of mode effective index (difference of 0.05 for metal stripe dimensions of *w* = 100 *nm* and *h* = 20 *nm*) that makes TiN a favorable material for such a photodetector. Usually, the lower the negative real part of permittivity, the longer the electric field penetration depth into the metal. However, in the proposed arrangement all the power is absorbed in a very thin metal stripe and very close to the metal-semiconductor interface, thus, most of the carriers can participate in a transition without experiencing any scattering with other carriers.

Our design takes advantages of two SPP modes that interact with the small volume metal stripe, thus more electrons will interact with the electro-magnetic energy giving rise to the higher efficiency of the IPE process, and consequence, higher photoemission. For a very thin metal stripe, there will be enhanced probability for hot carriers to cross the metal junction before thermalization^31^. In addition, the proposed design offers much better coupling efficiency from a photonic waveguide to a photodetector calculated at 98% for both aluminum^26^ and gold^25^ stripes compared to MIM and inverse-DLSPP arrangements where the coupling efficiencies were estimated at 50–60%. The improved coupling efficiency for the LR-DLSPP mode results from the similar mode profile of the photonic and fundamental LR-DLSPP modes. Higher coupling efficiency means that less light radiate through coupling due to the mode mismatch^29^ and more light decays in a non-radiative way by interaction with the metal giving rise to the hot carrier generation and improved photocurrent efficiency. Compared to the other designs with the MIM^19^ and inverse-DLSPP^18,28,29^ arrangements where the electrons have only a 50% probability to arrive at the metal-semiconductor interface, as they can travel either towards the interface or away from it^30^, in our proposed design this probability reaches 100% as the metal stripe is embedded in the semiconductor and all interfaces will participate in enabling transitions. Furthermore, as the metal stripe thickness for the LR-DLSPP arrangement is in the range from a few to tens of nanometers, the probability of inelastic collisions, and as a consequence, thermalization drops significantly for thinner metal stripes.

Non-radiative decay of SPP will produce hot electrons in the metal that will move towards the metal-semiconductor interface. As the metal stripe is entirely embedded in the semiconductor, most of the hot electrons will arrive to the interface. Taking into account a very thin metal stripe supporting a propagating mode that is in the range of 5–50 *nm*, and a mean free path of the electrons in metals (30–100 *nm*) most of the electrons will arrive at the interface without undergoing inelastic collisions. The hot electrons arriving at the metal-semiconductor interface with a kinetic energy exceeding the Schottky barrier Φ_*B*_ have a certain probability of passing over the barrier. To pass the barrier electrons have to conserve their energy and momentum tangential to the interface upon transmission through the barrier. Thus, metals with a lower Fermi level and with small band offset with the semiconductor material are more favorable. The impedance mismatch due to a large wavevectors contrast, between electrons in the metal and barrier can be alleviated at the corners of the metal stripe where the electric field is highly localized. It was observed that a longer interaction length in the plasmonic waveguide boost the efficiency of the photodetector. Furthermore, the hot electrons are directed along the polarization of the excited SPP that makes transitions to the semiconductor more probable. For a very thin semiconductor layer the tunneling through the barrier should be considered for the hot electron energies that are below the Schottky barrier^15^.

With decreasing metal thickness, the probability of generating hot electrons via intraband transition increases. As the plasmonic wavevector establishes the light wavevector the smaller fraction of the field is in the interior of the metal, which lowers the contributions of the interband transitions. For metal films thinner than 10 *nm* the probability of intraband transitions arises that in turn allow generation of hotter electrons for some metals like copper and gold^11^.

## Discussion

### Optical properties of TiN

To be considered as a metal for a plasmonic photodetector, TiN should show a good “metallic” behavior in the telecom wavelength range allowing guiding of plasmonic modes^34–37^. To determine the optical properties of TiN which was deposited by sputtering, variable angle spectroscopic ellipsometry measurements were performed on the 30 *nm* thick TiN films to obtain the optical constants (see Methods section).

**Figure 4:**
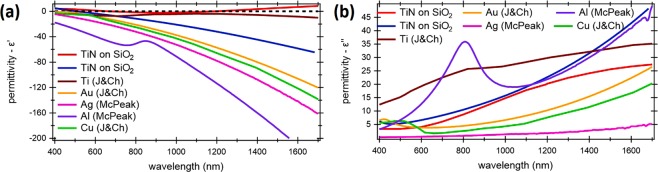
(**a**) Real and (**b**) imaginary part of permittivities for common metals with TiN fabricated under different deposition conditions (red and blue curves) (see Methods section)).

As can be seen from Fig. 4, the magnitude of the real part of TiN is smaller than that of other metals and the imaginary part of TiN is larger compared to other metals. Thus, TiN provides higher absorption efficiencies over a wider wavelength range that can enhance hot electron generation (Fig. 3). It is expected that losses exceeding 3–4 *dB*/*μm* are achievable with TiN. A smaller real part of the permittivity usually means a higher field penetration length into the metal. However, in the proposed design this effect is diminish through the LR-DLSPP arrangement where a very thin metal stripe is used.

### Electrical properties of TiN-Si contacts

**Figure 5:**
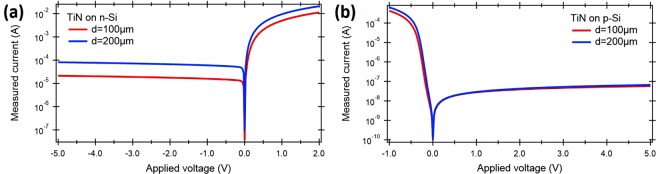
Dark I-V characteristics of the fabricated TiN-Si junction for different TiN contact areas - diameter *d* = 100 *μm* and *d* = 200 *μm* for (**a**) n-doped and (**b**) p-doped Si respectively.

Figure 5a,b depict the I-V dark measurements under dark current conditions for 30 *nm* of TiN deposited on n-Si and p-Si, respectively. The TiN is covered by a Ti/Au contact metal in the form of disks of different diameters and annular contact regions and then etched into devices of different sizes (see methods section). The dark current of Schottky diode is expressed by

1 $$I=S{A}^{\ast }{T}^{2}\,\exp (\frac{e{{\rm{\Phi }}}_{B}}{kT})\,[\exp (\frac{eV}{kT})-1]$$

where *S* is the contact area, A^*^ is the effective Richardson constant, Φ_*B*_ Schottky barrier height, and *V* is the applied voltage. As it can be observed from Fig. 5, the smaller contact area results in a smaller dark current. However, this behavior is better pronounced for the TiN on n-doped Si (Fig. 5a). The dark current for the TiN on p-doped Si is much lower compared to n-doped Si as a result of a higher Schottky barrier height (Fig. 5b) that limits the carriers flow from TiN to Si.

**Figure 6:**
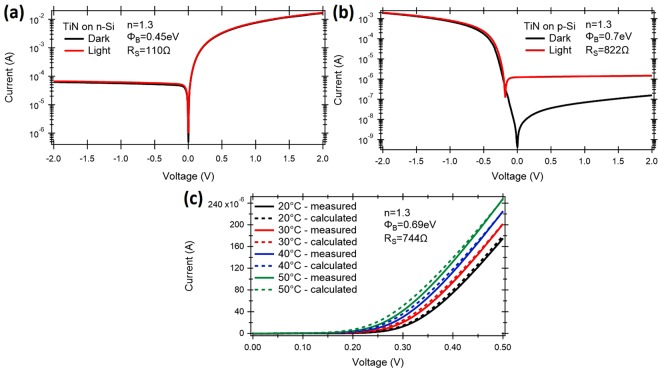
Opto-electronic characterization of the integrated Schottky photodetector. I-V measurements for TiN on (**a**) n-Si and (**b**) p-Si substrates without illumination and under white light illumination. (**c**) Temperature dependent electrical characterization of the TiN-(p-Si) contact. TiN contact diameter was *d* = 200 *μm*.

The Schottky barrier height was calculated for the TiN on n-doped Si (Fig. 6a) and p-doped Si (Fig. 6b). For p-Si (Fig. 6b) the device shows expected rectifying behavior with the forward bias region limited by the series resistance of the contact (*R*_*S*_ = 822 Ω) and dark current in order of 8.1 *nA* for reverse bias of 0.1 *V*. After illumination with visible light, a photocurrent of 1.24 *μA* was measured for reverse bias of 0.1 *V*. The ideality factor for this device was calculated at *n* = 1.3. On the contrary, for n-Si (Fig. 6a) the series resistance of the contact was calculated at *R*_*S*_ = 110 Ω and dark current of around 0.46 *μA* for a bias voltage of −0.1 *V*. After illumination, the photocurrent of 0.51 *μA* was measured. The ideality factor was calculated again at 1.3. For the TiN-(p-Si) contact, the device operates in photoconductive mode, where a higher optical signal affects primarily the reverse bias region, since the photogenerated process acts as an external current source added on top of the leakage (dark) current of the diode. As expected from the Schottky diode thermal dependence, the reverse current across the device increases with temperature due to enhanced thermionic emission of metal electrons into the silicon. In this operation mode, the variations of reverse leakage current are reflected to the forward bias region (Fig. 6c). Using the experimental data, the electrical parameters of the Schottky contact were extracted. The Arrhenius plot (*I*_0_/*T* vs. 1/*T*), where *I*_0_ is the leakage current and *T* is temperature, was used for extracting the barrier height Φ_*B*_, ideality factor *n*, series resistance *R*_*S*_ and the effective Richardson constant A^*^ ^38^. Thus, the potential barrier height at the TiN-(p-Si) interface, the ideality factor and the series resistance were found to be Φ_*B*_ = 0.69 *eV*, *n* = 1.3 and *R*_*S*_ = 744 Ω respectively, while Richardson constant was calculated at A^*^ = 120.15 *A*/*cm*^2^*K*^2^.

## Probability of hot electron transfer in TiN-based Schottky photodetector

TiN offers superior performances for hot carriers generation due to enhanced absorption efficiency and increased electron mean free path^39,40^. It ensures an increase in the number of hot electrons reaching the metal-semiconductor interface due to a longer effective mean free path compared to Au (Table 1)and lower carrier concentration than Au that diminish the energy loss of hot carriers due to inelastic scattering^34,35^. Another way to increases the hot carrier probability for transfer through the metal-semiconductor interface is to use metals with lower Fermi level. In this way, the cone of allowed wave vectors of hot electrons which can be injected into semiconductor increases as $si{n}^{2}\theta ={k}_{max}^{2}/{k}_{F}^{2}$, where *k*_*F*_ is the Fermi wave vector and *k*_*max*_ is the maximal *k*-vector that will allow transport of electrons from the metal to the semiconductor. Thus, the probability of internal photoemission of a hot electron which is generated by a photon with energy $\hslash \omega$ is given by:

2 $${P}_{0}(\hslash \omega )=\frac{1}{2}\frac{{E}_{F}}{\hslash \omega }\frac{{m}_{S}}{{m}_{0}}{(\frac{\hslash \omega -{{\rm{\Phi }}}_{B}}{{E}_{F}})}^{2}$$

where *m*_0_, *m*_*S*_ are the mass of the electron in the metal and the effective mass of the electron in the semiconductor respectively, and Φ_*B*_ is the Schottky barrier height between metal and semiconductor. The Fermi energy is defined as:

3 $${E}_{F}=\frac{{\hslash }^{2}{k}_{F}^{2}}{2{m}_{0}}=\frac{{\hslash }^{2}}{2{m}_{0}}{\mathrm{(3}{\pi }^{2}n)}^{\mathrm{2/3}}$$

where *n* is the carrier concentration. From the Drude fits to the dielectric function of Au and TiN (Fig. 4), the bulk plasmon frequencies of 8.9 *eV* for Au and 7.2 *eV* for TiN were obtained. The bulk plasma frequency *ω*_*p*_ depends on the carrier concentration and the effective mass of the electron in the metal:

4 $${\omega }_{p}^{2}=\frac{n{e}^{2}}{{m}_{0}{\varepsilon }_{0}}$$

where *e* is the elementary charge and *ε*_0_ is the permittivity of free space. Thus, the lower the plasma frequency, the lower the carrier concentration and consequently the lower the Fermi energy. As a result, the TiN has a much lower Fermi energy of 4.0–4.3 *eV* compared to the other common metals (Table 1).

**Table 1:** Properties of common metals used in the Schottky barrier photodetectors.

	Au	Ag	Al	Cu	Ti	TiN
Φ_*Bn*_[*eV*]	0.79–0.82	0.59–0.62	0.6–0.69	0.54–0.57	0.5	0.45
Φ_*Bp*_[*eV*]	0.32	0.43–0.46	0.42	0.37–0.4	0.61	0.7
Work function [*eV*]	5.1	4.2	4.1	4.6	4.33	4
Fermi energy level [*eV*]	5.51	5.48	11.63	7	—	4.2–4.3
Carrier free mean path [*nm*]	38	53	19	40	—	45–50

Thus, the escape cone can increases for at least 20 % compared to Au what enhance the transmission probability from the metal to semiconductor. This is proper for the assumption of constant density of states in the metal in the vicinity of Fermi level. However, in the proposed photodetector’s concept, the electric field is enhanced at all 4 metal stripe corners that makes the density of states in valence band of silicon higher, further enhancing the probability of hot carriers to be transferred through the barrier without reflection from the metal-semiconductor interface. Introducing the surface roughness between metal and semiconductor enhances the transmission probability (and thus injection efficiency) of hot electrons across the Schottky barrier. Up to an order of magnitude relative to the smooth interface at wavelength of 1550 *nm* enhancement can be achieved^27^.

## Signal-to-noise-ratio

Apart from the responsivity, another important figure of merit of the photodetector is the signal-to-noise ratio (SNR)^41^ defined as

5 $$SNR={i}_{signal}^{2}/{i}_{noise}^{2}$$

where *i*_*signal*_ and *i*_*noise*_ are the signal and noise currents, respectively. It is highly desired to enhance the signal while keeping the noise at low level. One solution to achieve a high SNR is by reducing the dimensions of the active Schottky junction area. Furthermore, the Schottky barrier between metal and semiconductor should be as close as possible to the optimal value of ~$0.697\,eV$ at telecom wavelength of 1550 *nm* (~$0.8\,eV$) that is calculated from equation^41^

6 $${{\rm{\Phi }}}_{{B}_{opt}}=h\nu -\frac{4kT}{e}$$

Thus, the Schottky barrier height between TiN and p-doped Si being calculated at Φ_*B*_ = 0.69–0.70 *eV* based on our measurements is perfect when compared with the optimal value of Φ_*Bopt*_ = 0.697 *eV* for an ideal diode^41^. The proposed photodetector collects the light from a photonic waveguide and concentrates it into a small metal stripe with a maximum concentration located at four corners of the stripe, thus providing high responsivity and low noise.

## Quantum efficiency and responsivity

For a thin metal stripe embedded into a semiconductor, the maximum number of possible trips for hot carriers into a metal film before its energy is reduced to *E*_*n*_ = Φ_*B*_ is given by^31,42^:

7 $$n=\frac{L}{t}\,\mathrm{ln}\,\frac{h\nu }{{{\rm{\Phi }}}_{B}}$$

where *t* is metal thickness, *L* - the attenuation length of hot carriers, i.e., the average distance over which a carrier can travel before experiencing a collisions and a reduction in energy, *hν* is a photon energy and Φ_*B*_ is a Schottky barrier. A carrier that is not emitted over the Schottky barrier is reflected toward the metal where it eventually reaches the barrier again and thus has another chance of emission. The total probability of photoemission is therefore the sum of the probabilities of carriers that have reflected off the barrier 0 to *n* times:

8 $$P({E}_{0})={P}_{0}+\mathrm{(1}-{P}_{0}){P}_{1}+\mathrm{(1}-{P}_{0}\mathrm{)(1}-{P}_{1}){P}_{2}+\,\mathrm{...}\,+{P}_{n}\prod _{k=0}^{n-1}\mathrm{(1}-{P}_{k})$$

where

9 $$P({E}_{k})=(1-\frac{{{\rm{\Phi }}}_{B}}{{E}_{k}});\,{\rm{for}}\,{E}_{k}={E}_{0}\,\exp (\,-\,kt/l) > {{\rm{\Phi }}}_{B}$$

The internal quantum efficiency for a thin-film metal embedded into a semiconductor, i.e., double-barrier, is calculated using the double-barrier emission probability:

10 $${\eta }_{i}=\frac{1}{h\nu }{\int }_{{{\rm{\Phi }}}_{B}}^{h\nu }\,P({E}_{0})d{E}_{0}$$

The double-barrier refers here to a metal film embedded into semiconductor thus forming a Schottky contact along two metal-semiconductor interfaces, as shown schematically in Fig. 2(b). Compare to it, a single barrier refers to the situation as presented in Fig. 2(a) where hot electrons can only cross a barrier that is in direct contact with a semiconductor. Finally, the responsivity *R* of the photodetector is calculated from:

11 $$R={\gamma }_{c}\mathrm{(1}-\exp (\,-\,\alpha l))\frac{q{\eta }_{i}}{h\nu }$$

where *γ*_*c*_ is the coupling efficiency of the photonic mode into LR-DLSPP mode, *hν* is photon energy, *α* is the attenuation constant of the plasmonic mode, *l* is the photodetector length, and *q* is the electronic charge. Thus, assuming TiN as a metallic stripe supporting the LR-DLSPP with the electron mean free path of *L* = 50 *nm* (*L* = 40 *nm* for gold), and two different thicknesses of TiN: *t* = 20 *nm* and *t* = 40 *nm* as presented in Fig. 6a, the number of possible round trips where calculated at *n* = 1.14 and *n* = 2.29. Taking a data for the Schottky barrier height as Φ_*B*_ = 0.45 for a n-doped Si (Fig. 6a), the emission probability was calculated at *P*(*E*) = 0.31 (for *t* = 20 *nm*) and *P*(*E*) = 0.26 (for *t* = 40 *nm*), and the internal quantum efficiency as *η*_*i*_ = 0.39 and *η*_*i*_ = 0.31 for *t* = 20 *nm* and *t* = 40 *nm* thick TiN metal stripe, respectively. From Fig. 3, the mode power attenuation can be calculated at *α* = 0.088 *μm*^−1^ for *t* = 20 *nm* and *α* = 0.417 *μm*^−1^ for *t* = 40 *nm*. Assuming a photodetector length of *l* = 20 *μm* and a coupling efficiency of *γ*_*c*_ = 92 % for *t* = 20 *nm*, and *γ*_*c*_ = 90 % for *t* = 40 *nm*, the responsivity was calculated at *R* = 0.37 *A*/*W* and *R* = 0.35 *A*/*W* for *t* = 20 *nm* and *t* = 40 *nm* thick TiN stripe, respectively. It has to be mentioned that no one parameter was optimized here to maximize the performance of the photodetector. For example, an increase of the TiN metal stripe width from *w* = 100 *nm* to *w* = 150 *nm* for a metal thickness of *t* = 20 *nm* and for the same waveguide dimensions will cause an increase in the photodetector responsivity to *R* = 0.5 *A*/*W*. By optimizing the plasmonic waveguide and integration with graphene^43–46^, a responsivity exceeding 1.0 *A*/*W* is expected^18,47^. When compared with ref.^29^ where a 100 *nm* thick Au film was deposited on top of the waveguide, the internal responsivity of *R* = 0.013 *A*/*W* was calculated. It is very consistent with the measured data presented in this paper where a responsivity of *R* = 0.0125 *A*/*W* was measured.

## Photodetector band diagram

**Figure 7:**
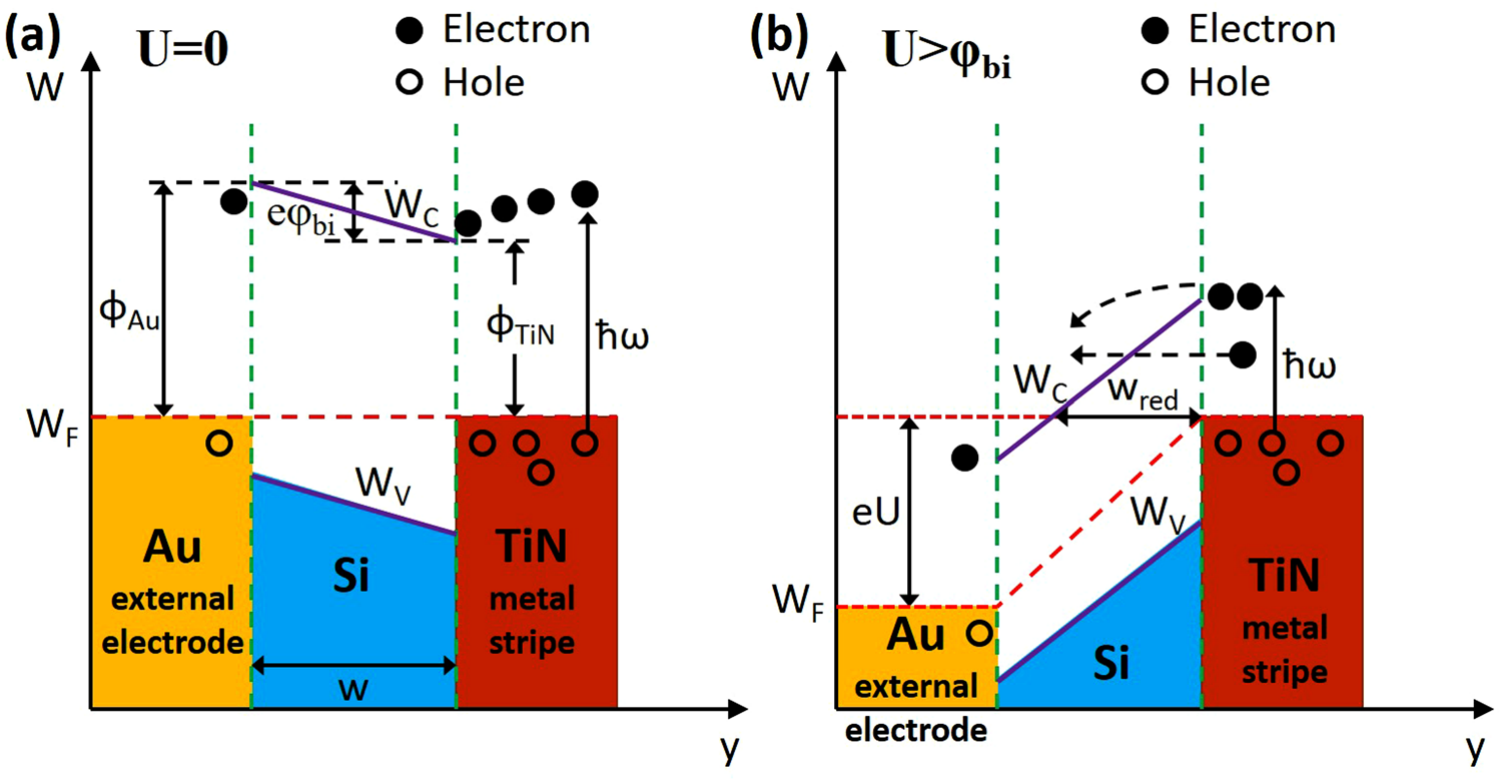
Energy band diagram of the Au-Si-TiN junction in (**a**) thermal equilibrium, no bias voltage and (**b**) nonequilibrium under applied forward bias voltage *V*, positive in the Au TiN direction.

For optimum operation conditions, i.e., low dark current and high photocurrent, the photodetector band diagram needs to be discussed. In our presented arrangement, the light coupled to the photodetector excites plasmonic LR-DLSPP with the SPPs propagating on both sides of the metal stripe and dissipating its energy entirely at the metal stripe - Si interfaces. The second electrode is placed outside of the waveguide on top of the Si rib. As the LR-DLSPP mode is bound to the metal stripe, the second electrode can be placed very close to the waveguide/photodetector without disturbing the propagating mode. This arrangement forms a MSM photodetector. The absorbed plasmonic wave creates hot electrons in the thin metal stripe that have an increased probability of crossing the potential barrier at the metal stripe - semiconductor interface. In the asymmetric MSM arrangement where both metal electrodes are from different metals, the built-in potential difference *φ*_*bi*_ across the silicon is created as a result of different Schottky barrier heights between the metals and semiconductor^42,48^. This impedes electron emission from the metal stripe into the silicon, and no significant current flow can be observed (Fig. 7a).

When a voltage is applied between electrodes with a positive potential at the external electrode exceeding the built-in potential difference *φbi*, the photoemission from the metal stripe is enabled that is on zero potential (Fig. 7b). This leads to a generation of photocurrent from the external electrode to the metal stripe side that depends on optical power coupled to the waveguide/photodetector. For the operation under low dark current conditions, the asymmetric metal arrangement allows the achievement of a reasonably high built-in potential difference across the silicon so that the dark current can be significantly suppressed under low operation voltages. A narrow metal stripe suppresses the dark current that depends on the area of the Schottky barrier contact. As a result, further reduction in a dark current can be achieve by using even narrower metal stripes while the absorption into metal, i.e., hot carrier generation, can be kept high by placing the metal stripe into the semiconductor ridge. This placement will disturb the propagating mode giving rise to the absorption in the metal. At the same time, the enhanced generation of hot electros in the metal stripe entirely embedded in the semiconductor enhances the photocurrent. As a result, the proposed photodetector arrangement is able to operate under low dark current conditions while the photocurrent can be highly enhanced compared to the previously presented configurations.

## Operation bandwidth

The small footprint associated with strong absorption of the plasmonic mode decreases the device capacitance such that the MSM bandwidth is not limited by RC time constant but rather by the transit time between electrodes^49^. The transit-time bandwidth *f*_*t*_ of the photodetector is proportional to the saturated drift velocity *v*_*c*_ in the semiconductor and inversely proportional to the distance between the contact electrodes *d*:

12 $${f}_{t}=0.45\frac{{v}_{c}}{d}$$

Assuming a reasonable carrier saturation velocity of $6\times {10}^{6}\,cm/s$ that is smaller than presented in a literature (1.1−1.4 × 10^7^*cm*/*s*)^50,51^, and a distance between electrodes of *d* = 400 *nm*, the transit-time bandwidth exceeding *f*_*t*_ = 67.5 *GHz* can be achieved. On the contrary, placing a metal stripe closer to the ridge wall (Figure Fig. 1b) will decrease the spacing between metal stripe and second electrode placed outside a photodetector. Thus, the transit time for carriers reaching a second electrode can be additionally decreased. As a consequence, the photodetector bandwidth can be increased. By reducing the spacing between both electrodes, i.e., metal stripe and the electrode placed outside the waveguide to 200 *nm*, the bandwidth can exceed 135 *GHz*. Furthermore, displacing the metal electrode inside a ridge will enhance the absorption in a metal stripe resulting in more hot carriers being generated inside the metal that can contribute to the photocurrent. As a result, the internal quantum efficiency will increase.

## Conclusion

Here we have proposed a novel plasmonic Schottky photodetector that takes full advantage of a metal stripe embedded into a semiconductor giving rise to the enhanced transmission probability of hot electrons from the metal to the semiconductor. Furthermore, it provides a coupling efficiency of the photonic mode to the photodetector that can exceed 90 %. As the metal stripe is very thin, much below the electron-mean-free path for metals, most of the hot electrons will participate in transmission to the semiconductor giving rise to an external quantum efficiency and responsivity that can exceed 1.0 *A*/*W*. Furthermore, it was shown that TiN is a perfect metallic material for the plasmonic photodetector as it provides higher electron-mean-free path and lower Fermi energy compared to most of the metals. Measurements showed that a Schottky barrier height of 0.69–0.70 *eV* exists between TiN and p-doped Si that ensures maximum SNR at 1550 *nm* wavelength calculated theoretically at 0.697 *eV*. Finally, TiN is a CMOS-compatible material that enable easy integration with existing CMOS technology. As a result, the proposed photodetector and TiN as a plasmonic material have the potential to overcome the existing responsivity and speed limitations of presently available photodetectors and become key component of future efficient and high-speed optical transmission systems.

## Methods

### Fabrication and optical characterization of TiN on Si

Thin 30 *nm*-thick films of TiN were deposited on n-Si/p-Si substrates by DC reactive magnetron sputtering from a 99.99% titanium target in an Argon-Nitrogen environment. To achieve a “metallic” TiN (blue curve in Fig. 4a,b), the deposition rate and substrate temperature were kept constant at 1.38 *nm*/*min* and 150 °C respectively. For TiN films deposited by atomic layer deposition resulted in oxygen incorporation in the film (red curve in Fig. 4(a,b)), the TiN exhibits “dielectric” properties over wavelength below 670 *nm* and above 1190 *nm*. In the wavelength range of 670–1190 *nm* the material shows poor “metallic” behaviour with the minimum real part of permittivity *ε*_*r*_ = −2.2 at wavelength of 930 *nm*. After deposition, the optical constants of the TiN films were derived from Spectroscopic Ellipsometry measurements performed using a J. A. Woollam M2000 variable angle spectroscopic ellipsometry system. This ellipsometry system was equipped with a rotating compensator and a high speed CCD camera. Measurements were performed at room temperature over a spectral range of 400–1700 *nm* and the dielectric functions were fitted to the Drude-Lorentz model^36,37^ (Fig. 4). After that, the results were compared with other metals commonly used as metal electrodes supporting a propagating plasmonic mode. The values of permittivity for gold, silver, aluminum, copper and titanium were taken from literature^34–37^.

### FEM simulations

The proposed modulator geometry was investigated using two-dimensional finite element method (FEM) simulations at the telecom wavelength of 1550 *nm* using commercial softwares COMSOL and Lumerical. The FEM is a well know technique for numerical solution of partial differential equations or integral equations, where the region of interest is subdivided into small segments and the partial differential equation is replaced with a corresponding functional. In the calculations, the refractive indexes of the Si waveguide and the SiO2 substrate was taken as *n*_*Si*_ = 3.48 and ${n}_{Si{O}_{2}}$ = 1.45, respectively. The Si ridge dimensions were kept constant at w = 380 *nm* and h = 200 *nm*, while rib thickness was kept at t = 140 *nm*. To compare absorption losses and mode effective index two different metals were used, Au and TiN, and two different metal dimensions, i.e., w = 100 nm, h = 20 nm, and w = 150 nm, h = 40 nm. The refractive index of gold (Au) and titanium nitride (TiN) was taken as *n*_*Au*_ = 0.52 + 10.74 *i* and *n*_*TiN*_ = 2.54 + 7.84 *i*, respectively (Fig. 4).

### Electrical characterization of TiN-Si contacts - I-V measurements

**Figure 8:**
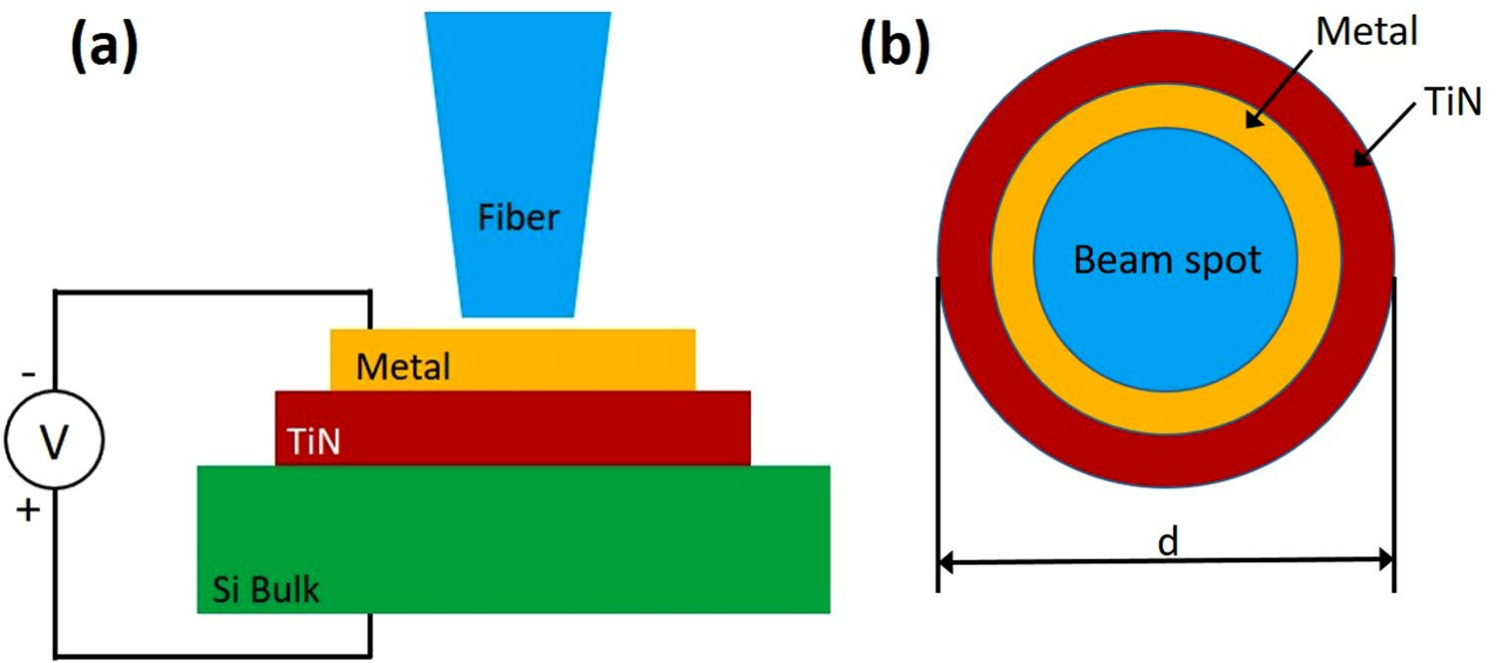
I-V measurement setup: (**a**) cross-section and (**b**) top view of the sample under measurement.

To characterize the electrical properties of TiN-Si contacts, we first measured a current-voltage (I-V) characteristic of the Schottky contact for both n-doped (*n* = 2–4 Ω*cm*) and p-doped (*n* = 10–20 Ω*cm*) silicon. The TiN thickness was kept constant at *h* = 50 *nm* for which a sheet resistance was measured as 50 Ω/*sq*. (*ρ* = 2.5 10^4^ Ω*cm*) while its diameter changed from *d* = 100 *μm* to *d* = 200 *nm* (Fig. 8). A ring shaped Au structure was formed the top contact on TiN/p-Si device. The substrate was used as the bottom contact. The device was probed under microscope and illuminated by a 1550 *nm* wavelength laser during measurements. An Er-doped fiber amplifier that can output up to 20 *mW* optical power was used as the infrared light source. The device was exposed to the infrared light by bringing a lens ended fiber to the close proximity of the top of the TiN layer surrounded by the ring shaped Au contact. Current-voltage measurements were done by using a Keithley Sourcemeter. Temperature dependent current-voltage (I-V) measurements are conducted in a similar configuration. The sample was placed on a metal chuck and probed under a microscope. The temperature of the metal chuck was controlled by a thermoelectric cooler (TEC) and Keithley 2510 TEC Sourcemeter. I-V measurements were done at 20 °C, 30 °C, 40 °C and 50 °C.
